# Identification of Major Active Ingredients Responsible for Burn Wound Healing of *Centella asiatica* Herbs

**DOI:** 10.1155/2012/848093

**Published:** 2012-12-30

**Authors:** Fang Wu, Difei Bian, Yufeng Xia, Zhunan Gong, Qian Tan, Jiaojiao Chen, Yue Dai

**Affiliations:** ^1^State Key Laboratory of Natural Medicines, Department of Pharmacology Chinese Materia Medica, China Pharmaceutical University, 24 Tong Jia Xiang, Nanjing 210009, China; ^2^Center for New Drug Research and Development, College of Life Science, Nanjing Normal University, Nanjing 210024, China; ^3^Department of Burns and Plastic Surgery, Nanjing Drum Tower Hospital, Medical School of Nanjing University, Nanjing 210008, China

## Abstract

*Centella asiatica* herbs have been prescribed as a traditional medicine for wound healing in China and Southeast Asia for a long time. They contain many kinds of triterpenoid compounds, mainly including glycosides (asiaticoside and madecassoside) and corresponding aglycones (asiatic acid and madecassic acid). To identify which is the major active constituent, a comprehensive and comparative study of these compounds was performed. *In vitro*, primary human skin fibroblasts, originating from healthy human foreskin samples, were treated with various concentrations of asiaticoside, madecassoside, asiatic acid, and madecassic acid, respectively. Cell proliferation, collagen synthesis, MMP-1/TIMP-1 balance, and TGF-**β**/Smad signaling pathway were investigated. *In vivo*, mice were orally administered with the four compounds mentioned above for two weeks after burn injury. The speed and quality of wound healing, as well as TGF-**β**_1_ levels in skin tissues, were examined. Interestingly, in contrast to prevalent postulations, asiaticoside and madecassoside themselves, rather than their corresponding metabolites asiatic acid and madecassic acid, are recognized as the main active constituents of *C. asiatica* herbs responsible for burn wound healing. Furthermore, madecassoside is more effective than asiaticoside (*P* = 0.0446 for procollagen type III synthesis *in vitro*, *P* = 0.0057 for wound healing speed, and *P* = 0.0491 for wound healing pattern *in vivo*, correspondingly).

## 1. Introduction

Burn injury is a dermal or other organic tissue injury mainly caused by thermal or chemical insults, and includes scalds, contact, and flame burns [1]. According to the report presented by World Health Organization (WHO) at the year 2002, it was estimated an annual casualty of 330,000 deaths was, directly or indirectly, related to burn injury [2]. 

Immediately after burn injury, skin responds to the affliction with a complex, however, well-orchestrated, wound healing process. The central zone of coagulation consists of irreversible cell and tissue necrosis due to direct thermal injury. Neutrophils and later monocyte-derived macrophages are attracted to the wound in response to a constellation of chemoattractants secreted from platelets in the hemostatic clot [3]. Growth factors, especially transforming growth factor (TGF), are released after the activation of macrophages by necrotic debris, and gradually tuning the inflammatory setting into a milieu that favors fibroblast proliferation and extracellular matrix (ECM) synthesis [4]. As a self-limited process, normal wound healing ends up with a remodeling phase characterized by apoptosis of myofibroblasts and macrophages, secretion of matrix metalloproteinases (MMPs), and tissue inhibitor of metalloproteinases (TIMPs) from fibroblasts, which finally recovers the texture and strength close to normal state [5].

**Figure 1:**
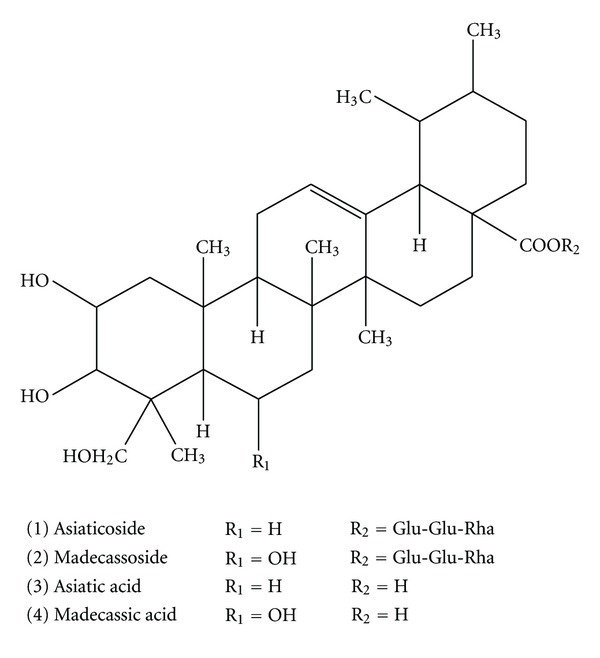
Chemical structures of asiaticoside, madecassoside, asiatic acid, and madecassic acid.

 Any derailment in the wound repair process would lead to a variety of ailments, including chronic wound healing [6], hypertrophic scar [7], and keloid [8]. Considered as a major threat to public health, abnormal wound repair also lays a heavy burden on the economy [9]. Therefore, attempts to facilitate wound repair have never been staggered. Unfortunately, current clinical application of antibiotics, single cytokines, and growth factors showed unsatisfactory therapeutic effects [10]. As a complementary alternative, *Centella asiatica* herbs have been used orally and topically to improve wound repair for years [11]. As shown in Figure 1, triterpenoid compounds occurring in *C. asiatica*, mainly including two glycosides (asiaticoside and madecassoside) and corresponding aglycones (asiatic acid and madecassic acid), are considered to be major ingredients with pharmacological activities [12]. Previous independent studies have showed that both asiaticoside and madecassoside could facilitate burn wound healing possibly via enhancing collagen I synthesis [13–15]. On the other hand, current studies on the two aglycones asiatic acid and madecassic acid focus on their proapoptotic and anti-inflammatory effects [16–19]. Hence, our group postulated that madecassoside, administered orally, might exert anti-inflammatory effects in collagen II-induced arthritis mice through its intestinal metabolic product madecassic acid but not itself [20], which was further supported by Won et al. [18]. Nevertheless, which is the main active constituent for burn wound healing effects of *C. asiatica* herbs; and how asiaticoside and madecassoside function after oral administration needs to be clarified.

Therefore, this study was designed to investigate the effects of the four major triterpene constitutes in *C. asiatica* as mentioned above on *in vitro* collagen synthesis and *in vivo* burn wound healing, simultaneously. A comprehensive comparison and analysis should enable us to understand the actual active constituents of *C. asiatica* herbs for burn wound healing.

## 2. Materials and Methods

### 2.1. Chemicals and Reagents

 Madecassoside (C_48_H_78_O_20_, MW: 975.12), asiaticoside (C_48_H_78_O_19_, MW: 959.12), madecassic acid (C_30_H_48_O_6_, MW: 504.70), and asiatic acid (C_30_H_48_O_5_, MW: 488.70) were generous gifts from Dr. Zhunan Gong, and the purities (≥98%) were determined by HPLC-ELSD. A voucher specimen (Gong 0703 : 1–4, resp.) was maintained in the Center for New Drug Research & Development, College of Life Science, Nanjing Normal University. Cell-Light EdU Apollo 567 DNA *in vitro* kit was purchased from RiboBio Co. Ltd. (Guangzhou, China). EasyScript First-Strand cDNA synthesis SuperMix and EasyTaq DNA Polymerase used for RT-PCR were products of TransGen Biotech (Beijing, China). ELISA kits for procollagen type I N-terminal propeptide (PINP) and procollagen type III N-terminal propeptide (PIIINP) detection were purchased from R&D Systems Inc. (Minneapolis, MN, USA). Anti-Smad 3, anti-phospho-Smad 3 and anti-glyceraldehyde-3-phosphate dehydrogenase (GAPDH) were purchased from Cell Signaling Technology (Beverly, MA).

### 2.2. Primary Cell Culture

Primary human skin fibroblasts were obtained from healthy foreskin samples of patients with ablative surgery via enzymatic digestion. All the experiments were conducted in accordance to the Declaration of Helsinki and were approved by the Ethics Committee of Zhongda Hospital (Nanjing, China). Cells were cultured in Dulbecco's modified Eagle medium (DMEM, Gibco, Grand Island, NY, USA) supplemented with 10% fetal bovine serum (FBS, HyClone, Logan, UT, USA), 100 U/mL streptomycin and 100 U/mL penicillin. All *in vitro* assays were conducted using cells between passage 2 and passage 6.

### 2.3. Cell Proliferation Assay

Fibroblasts (1 × 10^5^ cells/well) were seeded onto 96-well plates and incubated in full media overnight. Cells were synchronized with a 24 h serum-free treatment before the addition of 5-ethynyl-2-deoxyuridine (EdU) with or without the treatment of four triterpene compounds (1, 3, 10 *μ*M). After a 48 h-incubation, cells were fixed and permeabilized for EdU detection according to the manufacturer's protocol. Hoechst 33342 was applied to locate the nucleus. Cells were photographed using a fluorescence microscope (Olympus). The proliferation rate was calculated by normalizing the number of EdU-positive cells to the Hoechst 33342-stained cells in nine random fields. 

### 2.4. Reverse Transcription Polymerase Chain Reaction (RT-PCR) Analysis

 After treatment with varying concentrations of four triterpene compounds (1, 3, 10 *μ*M) for 24 h, cellular total RNA was extracted using TRIzol reagent (Invitrogen, Carlsbad, CA) and reverse-transcribed according to the EasyScript kit's protocol. Primers used for collagen type I, collagen type III, MMP-1, TIMP-1, TGF-*β*_1_, T*β*RI, T*β*RII, Smad 7, and *β*-actin were listed in Table 1. PCR products were separated by 1.2% agarose gel electrophoresis. A final concentration of 0.005% ethidium bromide was added to the gel for the visualization of PCR products in a BioRad UV chamber. Photos were taken and gene expressions were quantified by using Image J imaging software (US National Institute of Health, Bethesda, MD) via densitometry.

### 2.5. Quantitative Detection of Procollagen I and Procollagen III

The contents of procollagen I and procollagen III were determined by commercial kits, which were capable of detecting procollagen I N-terminal peptide (PINP) and procollagen III N-terminal peptide (PIIINP) via enzyme-linked immunosorbent assay (ELISA). Cells (2 × 10^5^ cells/well) were seeded in 48-well plates and incubated at 37°C with 5% CO_2_. Procollagen contents in supernatants were measured according to manufacturer's protocol after cells were treated with different concentrations of four triterpene compounds (1, 3, 10 *μ*M) for 24 h.

### 2.6. Western Blot Analysis

Cells, pre-incubated with different concentrations of four triterpene compounds (1, 3, 10 *μ*M) for 24 h, were collected and lysed on ice in cell lysis buffer for 30 min, and centrifuged for 5 min at 12,000 rpm. Fifty micrograms of protein samples were separated on 10% SDS-PAGE and later transferred to polyvinylidene difluoride (PVDF) membranes. The membranes were incubated with specific anti-Smad 3 (1 : 1000), anti-phospho-Smad 3 (1 : 1000) and anti-GAPDH (1 : 5000) primary antibodies at 4°C overnight, followed by incubation with HRP-conjunct secondary antibodies for 1 h. Protein bands were visualized through Luminata Crescendo Western HRP Substrate (Millipore, Billerica, MA), and the optical density of each band was determined by using Image J.

### 2.7. Animals

Male ICR mice, weighing between 18–22 g, were purchased from the experimental animal center of China Pharmaceutical University. Animals were housed in a room with a constant temperature (22 ± 1°C) and a 12-h light-dark cycle, and fed with standard diet and water *ad libitum*. All experiments were performed strictly under the guidance of the Ethical Regulations for Institutional Animal Care and Use of China Pharmaceutical University.

### 2.8. Burn Injury Model and Drug Administration

Full-thickness burn injury was conducted following a published protocol [21]. In brief, mice (*n* = 120) were anesthetized with pentobarbital (40 mg/kg, i.p.). Back hair was removed and direct contact between a brass rod (65 g, 1 cm in diameter) heated to 95°C and the back skin was kept for 9 seconds to induce full-thickness burn wound. Equal molar of four triterpene compounds were dissolved in distill water, and administered orally (6, 12, 24 mg/kg for asiaticoside and madecassoside; 3, 6, 12 mg/kg for asiatic acid and madecassic acid) for 14 consecutive days. Control group (*n* = 24) was handled in the same way except for administering distill water. Wound was photographed, and the areas were measured on days 0, 3, 7, 11, 14 by using Image J. At the same time points, the total wounds were biopsied and fixed in 10% formalin for further analysis.

### 2.9. Histological Assessment

Formalin-fixed tissue specimens were embedded with paraffin and sectioned serially at 5 *μ*m. Sections were stained with haematoxylin and eosin (H&E) for histologic analysis and were scored by professional histologists from a cluster of aspects including: (1) necrosis and ulcer; (2) edema and inflammatory cell infiltration; (3) granulation tissue formation; and (4) re-epithelialization. Moreover, Masson's trichrome stain was performed to establish a clearer vision on extracellular matrix (ECM) deposition in order to specify collagen amount and arrangement.

### 2.10. TGF-*β*_1_ Immunohistochemistry

Mouse wound tissue sections obtained at post-burn 7 and 14 days were sectioned serially at 5 *μ*m. Slides were incubated with anti-TGF-*β*_1_ primary antibody (Abcam, Cambridge, MA) for 1 h, and followed by development with DAB Envision System (DAKO, Ely, UK) according to the manufacturer's protocol.

### 2.11. Statistical Analysis

All data were expressed as mean ± standard deviation (SD). Differences between control and experimental groups were analyzed by one- or two-way analysis of variance followed by Dunnett's test, and *P* < 0.05 was accepted as statistically significant. All the calculations were performed using SPSS statistical software (SPSS, Chicago, IL).

## 3. Results

### 3.1. Effects of Four Triterpene Compounds on the Proliferation of Human Skin Fibroblasts

**Figure 2:**
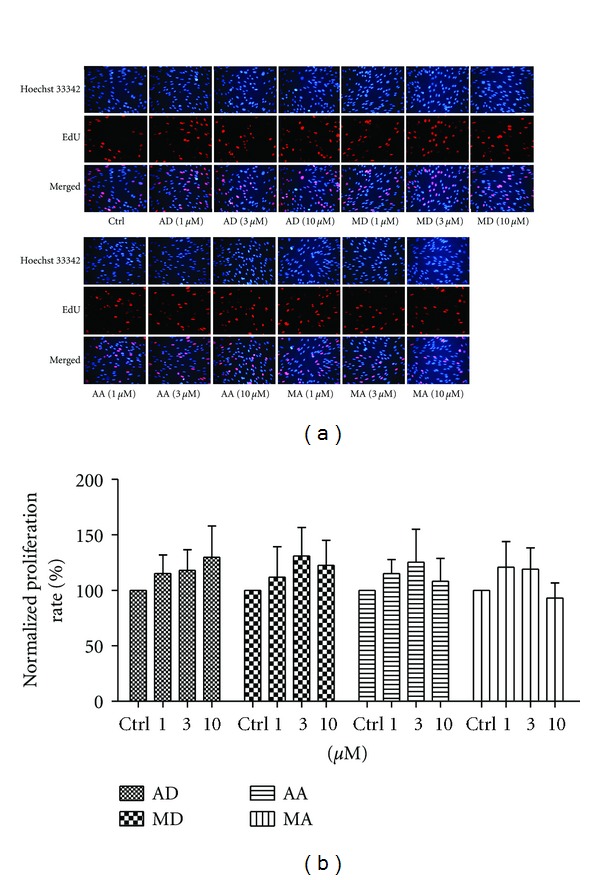
Effects of asiaticoside (AD), madecassoside (MD), asiatic acid (AA), and madecassic acid (MA) on the proliferation of huamn skin fibroblasts. (a) Representative pictures of immunofluorescence staining for EdU (red) and Hoechst 33342 (blue) in the nucleus of cells after treated with the four compounds for 48 h (magnification 250x). (b) Proliferation rates were calculated by normalizing the number of EdU-positive cells to the Hoechst-stained cells in nine fields at 250x magnification. Each column represents the mean ± S.D. from three independent experiments.

As fibroblasts played a pivotal role in wound healing, they were used for *in vitro* studies. During the granulation tissue formation period, ECM, mainly collagen type I and type III, was synthesized and secreted by fibroblasts. The proliferation of skin fibroblasts might be directly linked to faster ECM deposition and consequent accelerated wound healing. Therefore, we investigated the effects of four triterpene compounds on the proliferation of skin fibroblasts via EdU assay. As shown in Figure 2, all test compounds at concentrations (1, 3, 10 *μ*M) could not enhance cell proliferation, as compared with control group.

### 3.2. Effects of Four Triterpene Compounds on Collagen Synthesis of Human Skin Fibroblasts

**Figure 3:**
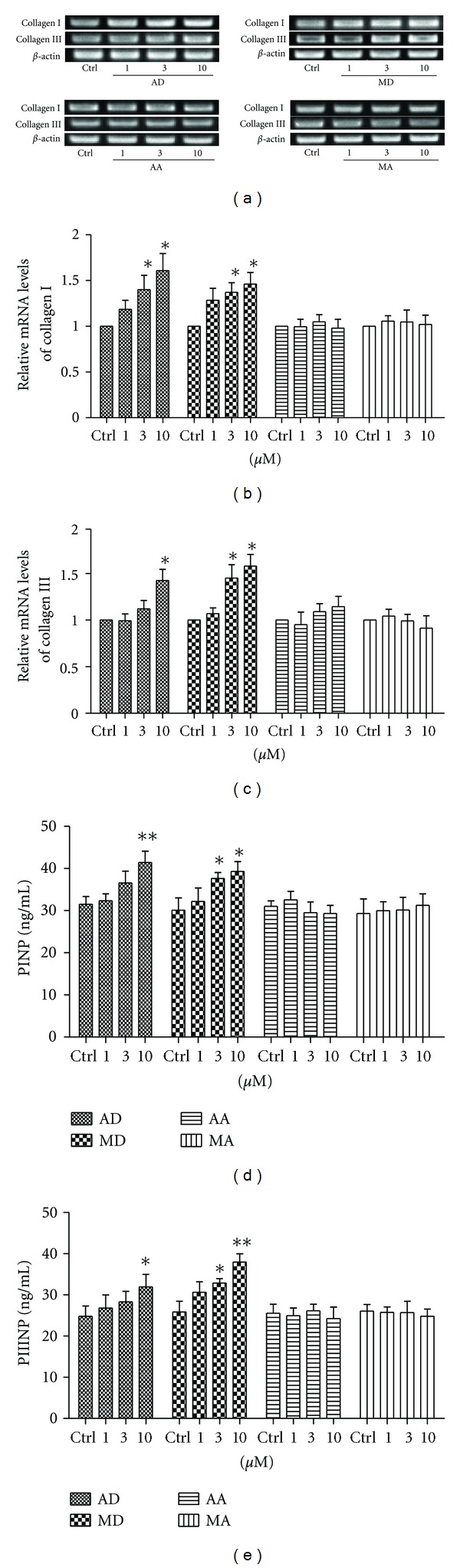
Effects of asiaticoside (AD), madecassoside (MD), asiatic acid (AA), and madecassic acid (MA) on collagen synthesis in human skin fibroblasts. The cells were treated with various concentrations of the four triterpene compounds for 24 h. ((a)–(c)) The mRNA expressions of collagen type I and type III were determined via RT-PCR assay. ((d), (e)) The cell supernatants were prepared and analyzed for protein expressions of procollagen I N-terminal peptide (PINP) and procollagen III N-terminal peptide (PIIINP) by ELISA. Each column represents the mean ± S.D. from three independent experiments. *P < 0.05, **P < 0.01 versus control.

Collagen, especially type I and type III collagen in skin tissue, is of great benefit to wound healing. Therefore, we investigated the effects of four triterpene compounds on collagen synthesis in skin fibroblasts. The results were exhibited in Figure 3. Both asiaticoside and madecassoside (3, 10 *μ*M) significantly elevated mRNA levels of collagen type I and type III in fibroblasts as determined by RT-PCR (Figures 3(a)–3(c)), as well as protein levels of procollagen type I and type III as detected by ELISA (Figures 3(d) and 3(e)). In contrast, neither asiatic acid nor madecassic acid could influence collagen synthesis in fibroblasts as compared with control. Furthermore, it is noteworthy that madecassoside was more effective than asiaticoside at 10 *μ*M (*P* = 0.0446). 

### 3.3. Effects of Four Triterpene Compounds on Collagen Degradation of Human Skin Fibroblasts

**Figure 4:**
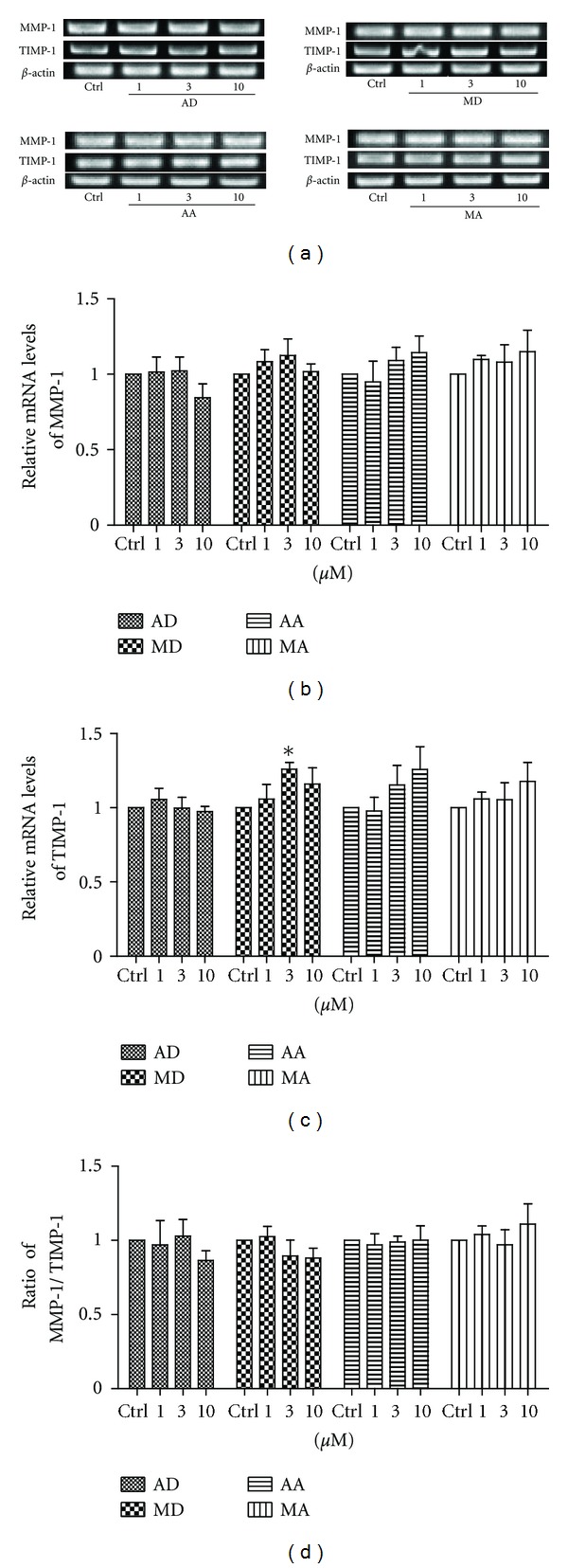
Effects of asiaticoside (AD), madecassoside (MD), asiatic acid (AA), and madecassic acid (MA) on the expressions of MMP-1 and TIMP-1 mRNA in human skin fibroblasts. The cells were treated with various concentrations of the four triterpene compounds for 24 h. ((a)–(c)). The mRNA expressions of MMP-1 and TIMP-1 were measured via RT-PCR assay. (d) The ratios of relative intensity between MMP-1 mRNA and TIMP-1 mRNA were calculated. Each column represents the mean ± S.D. from three independent experiments. *P < 0.05 versus control.

Collagen synthesis is not the only contributor to ECM deposition. During granulation tissue formation and consequent remodeling period, MMP-TIMP system plays a crucial role in collagen degradation as well. MMP-1, also known as collagenase I, could degrade various types of collagen, while TIMP-1 inhibits the activity of MMP-1. Therefore, both MMP-1 and TIMP-1 mRNA expressions were investigated via RT-PCR. As TIMPs bind with MMPs usually in stoichiometrical fashions of 1 : 1 or 2 : 2, the ratio of MMP-1 to TIMP-1 should be more intuitive to describe the pharmacological activities of four triterpene compounds on collagen degradation. As depicted in Figures 4(a)–4(d), although madecassoside (3 *μ*M) could significantly enhanced TIMP-1 mRNA expression, the four compounds failed to affect MMP-1/TIMP-1 ratio in human skin fibroblasts.

### 3.4. Effects of Four Triterpene Compounds on TGF-*β*/Smad Pathway of Human Skin Fibroblasts

**Figure 5:**
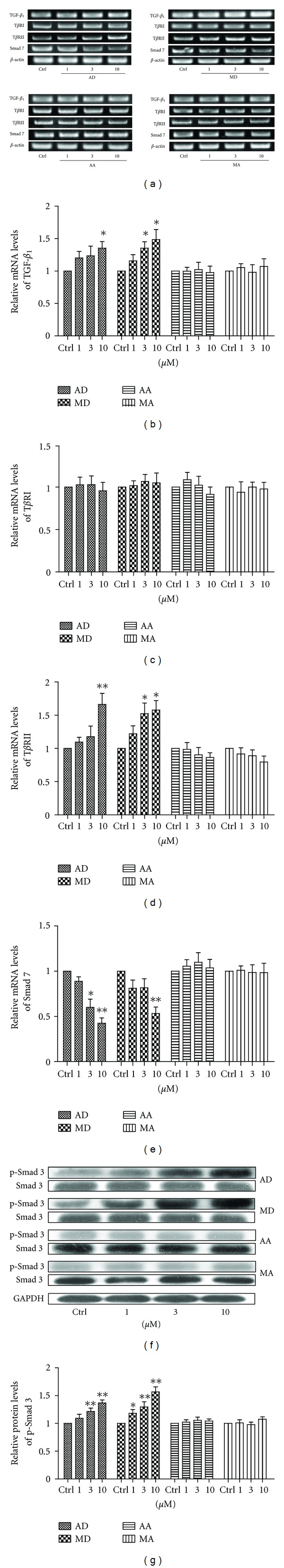
Effects of asiaticoside (AD), madecassoside (MD), asiatic acid (AA), and madecassic acid (MA) on TGF-*β*/Smad pathway in human skin fibroblasts. ((a)–(e)) The mRNA expressions of TGF-*β*1, T*β*RI, T*β*RII and Smad 7 were measured viaRT-PCR assay. ((f), (g)) The phosphorylation of Smad 3 was measured via Western blot assay. Each column represents the mean ± S.D. from three independent experiments. *P < 0.05, **P < 0.01 versus control.

TGF-*β*/Smad signal pathway, mainly including TGF-*β*_1_ and relevant receptors T*β*RI, II as well as Smad 3 and 7, is deeply involved in the activation, differentiation to myofibroblasts, and resultant collagen synthesis of fibroblasts. To recognize the mechanisms by which the triterpene constituents from *C. asiatica* accelerated collagen synthesis, the effects on TGF-*β*/Smad pathway of human skin fibroblasts were explored. The results were exhibited in Figure 5. Both asiaticoside (10 *μ*M) and madecassoside (3, 10 *μ*M) significantly increased TGF-*β*_1_ and T*β*RII mRNA expression, decreasing Smad 7 mRNA expression and elevating phosphorylation levels of Smad 3 in fibroblasts, while they had no influence on T*β*RI expression (Figures 5(a)–5(g)). In contrast, neither asiatic acid nor madecassic acid could influence TGF-*β*/Smad pathway in fibroblasts as compared with control. Furthermore, it should be noted that madecassoside was more effective than asiaticoside at 10 *μ*M (*P* = 0.0487), which was consistent with above results on collagen synthesis.

### 3.5. Effects of Four Triterpene Compounds on Burn Wound Healing in Mice

**Figure 6:**
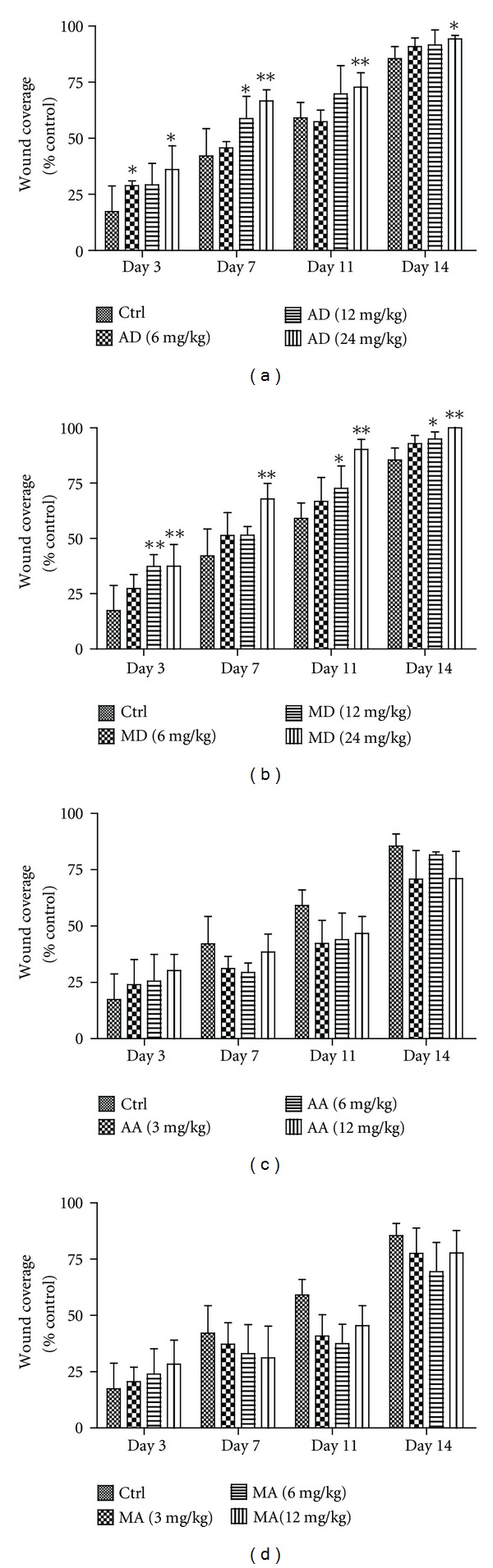
Effects of asiaticoside (AD), madecassoside (MD), asiatic acid (AA), and madecassic acid (MA) on the wound coverage of burn injury model. Various doses of the four triterpene compounds were orally administered after burn injury for two weeks. Wound coverage areas ((a)–(d)) were evaluated at indicated days. Data are shown as mean ± S.D. for each group (*n* = 5). *P < 0.05, **P < 0.01 versus control.

**Figure 7:**
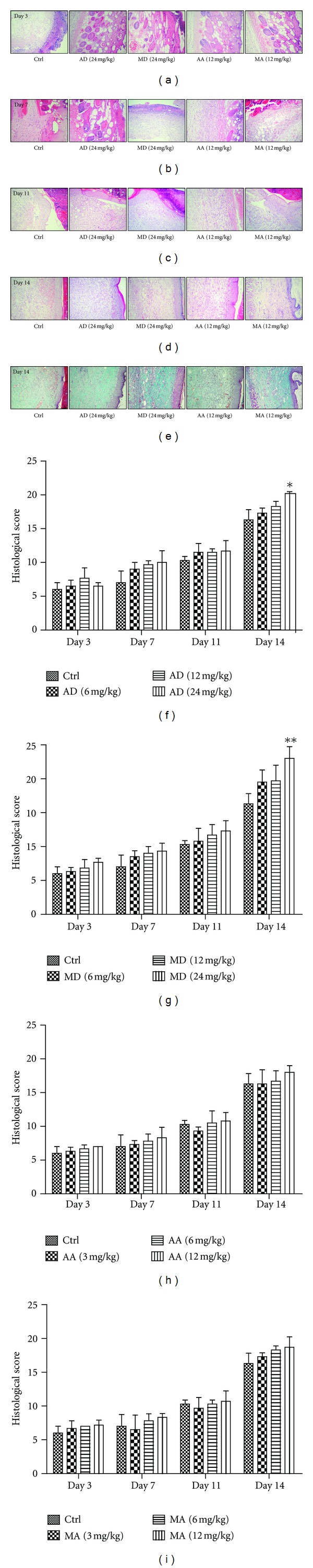
Effects of asiaticoside (AD), madecassoside (MD), asiatic acid (AA), and madecassic acid (MA) on histopathological changes in wound skin tissues. Various doses of the four triterpene compounds were orally administered after burn injury for two weeks. The skin samples were obtained at indicated days after burn injury, and stained by Haematoxylin and Eosin stain (H&E) and Masson's trichrome stain. The presented images of H&E stained tissues ((a)–(d)), and Masson's trichrome stain tissues (e) are taken from various groups at indicated days, magnification 100x. Histological assessments ((f)–(i)) were scored by professional histologist from a cluster of aspects as described. Data are shown as mean ± S.D. for each group. *P < 0.05, **P < 0.01 versus control.

*In vitro* assays described above showed that two glycosides asiaticoside and madecassoside, rather than their aglycones asiatic acid and madecassic acid, were able to concentration-dependently enhance collagen type I and type III synthesis mainly through activating skin fibroblasts via TGF-*β*/Smad pathway, and madecassoside showed more potent effect than asiaticoside (*P* = 0.0446). To verify this, a wound healing test was performed. As shown in Figures 6 and 7, both asiaticoside and madecassoside not only accelerated wound healing, but also result in a better wound healing pattern in a view of histological examination. Madecassoside (24 mg/kg)-treated group showed significantly better wound healing speed and wound healing results as compared with asiaticoside (24 mg/kg)-treated group (*P* = 0.0057 and *P* = 0.0491, correspondingly). 

**Figure 8:**
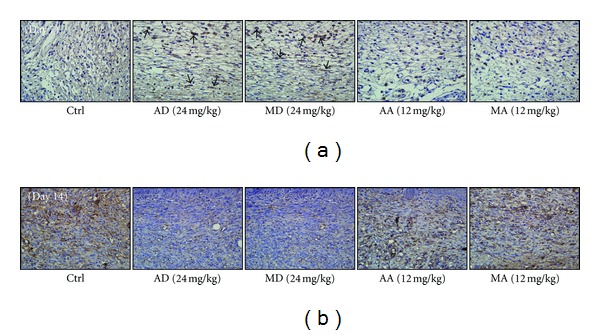
Effects of asiaticoside (AD), madecassoside (MD), asiatic acid (AA), and madecassic acid (MA) on TGF-*β*_1_ expression in wound skin tissues. Images of DAB-stained TGF-*β*_1_ were taken from various groups at Day 7 (a) and Day 14 (b) after injury, magnification 200x. Positive labeled macrophages and fibroblasts are indicated with arrows.

The immunohistochemical-stains of TGF-*β*_1_ (Figure 8) were also consistent to above results. At day 7 post-burn, there were dark stains in infiltrated macrophages and fibroblasts in two glycosides-treated groups. While at day 14 post-burn, DAB stains could be merely seen in glycosides-treated groups, which might be the consequence of faster maturation of granulation tissue. Furthermore, neither asiatic acid nor madecassic acid could facilitate burn wound healing.

## 4. Discussion

As a traditional herbal medicine in China and southeastern Asia for ages, *Centella asiatica* (Umbelliferae) was applicable for a wide range of indications, such as ulcers, leprosy, and psoriasis. However, promoting wound healing is its primary application [12, 22]. Aisaticoside, madecassoside, asiatic acid, and madecassic acid were reported as major constituents of *C. asiatica *with pharmacological activities [23]. Previous independent studies have disclosed that *C. asiatica* extract, together with two glycosides, namely asiaticoside and madecassoside, could facilitate wound healing either induced by surgical or thermal injuries [13, 15, 24]. However, whether asiaticoside or madecassoside is more effective in accelerating wound healing remains unknown. In the present study, we investigated the wound healing effects of four major constitutes of *C. asiatica* in a comprehensive and comparative way. Our *in vitro* assay results were in line with the prior reports that asiaticoside and madecassoside could elevate collagen synthesis via activating TGF-*β*/Smad signaling pathway. *In vivo* assay further confirmed that asiaticoside and madecassoside are entities with wound healing activities in *C. asiatica*, and madecassoside showed a faster healing speed under macroscopic observation and a better healing pattern from the histological aspect than asiaticoside, which is consistent to *in vitro* assay results (*P* = 0.0057 and *P* = 0.0491, correspondingly). Noteworthily, asiatic acid and madecassic acid, which are the metabolites of asiaticoside and madecassoside and were formerly assumed as active entities of *C. asiatica*, showed no wound healing activities either *in vitro* or *in vivo*, as compared to the control group.

Burn wound healing is composed of three overlapping stages, which are inflammation, granulation tissue formation and remodeling [5]. A series of coagulation cascade is initiated by burn injuries, which forms a platelet plug, later on a fibrin matrix. Served as a temporary scaffold for infiltrating cells, the fibrin matrix is replaced by granulation tissue and plays a role in keratinocyte migration. Macrophage, which secretes TGF-*β*, attracts fibroblasts from the wound edge or from bone marrow to the wound area [25]. There are three isotypes of TGF-*β*, namely TGF-*β*_1_, TGF-*β*_2_, and TGF-*β*_3_. Among which, TGF-*β*_1_ is closely related to wound healing [23]. Our *in vitro* RT-PCR assay showed that asiaticoside and madecassoside could elevate TGF-*β*_1_ mRNA expression in skin fibroblasts after the possibility of cell proliferation was excluded. Further *in vivo* observation via immunohistochemistry displayed dark stains of TGF-*β*_1_ in infiltrated macrophages and fibroblasts at day 7 post-burn in both asiaticoside- and madecassoside-treated groups, suggesting that asiaticoside and madecassoside might facilitate burn wound healing via inducing TGF-*β*_1_ expression.

TGF-*β* first binds to TGF beta type II receptor (T*β*RII), and later the heterodimer was recruited to TGF beta type I receptor (T*β*RI), and leads to the phosphorylation of T*β*RI. Receptor-regulated SMADs (R-SMADs), including Smad 2 and Smad 3, are then phosphorylated and translocated to the nucleus with the participation of co-SMAD, Smad 4. Smad 7, acting as an inhibitory Smad, competes with R-SMADs for receptor binding and thus abrogates TGF-*β* signaling transduction [26]. Under the stimulation of TGF-*β*_1_, fibroblasts proliferate and secrete extracellular matrix (ECM), mainly collagen type I and type III, and also partially differentiate into myofibroblasts which contribute to an accelerated closure by contraction [27]. Therefore, we conducted *in vitro* RT-PCR assay detecting mRNA expressions of T*β*RI, T*β*RII and Smad 7. Asiaticoside and madecassoside (10 *μ*M) significantly elevated mRNA levels of T*β*RII and inhibited Smad 7 mRNA expression, while all four compounds were lack of significant effect on the expression of T*β*RI, a constitutive expressed membrane receptor. The phosphorylation levels of Smad 3 were also elevated in asiaticoside- and madecassoside-treated groups, indicating that the two compounds could activate TGF-*β*/Smad signaling pathway.

After translocated to nucleus, heteromeric Smad complex should bind to SMAD binding element at the promoter site of collagen type I and type III with transcriptional coactivators and corepressors like p300 and CBP to regulate gene expression [28]. For wound healing, collagen type III was synthesized at first as a provisional matrix, and later been substituted with collagen type I during remodeling phase [5]. Elevated procollagen type I and type III expression both at mRNA and protein levels have been observed in both glycosides-treated groups. Furthermore, at 10 *μ*M, madecassoside was significantly more potent than asiaticoside in elevating procollagen type III synthesis, which could account for a faster and better wound healing in our burn wound healing model (*P* = 0.0446). 

Matrix metalloproteinases (MMPs) and tissue inhibitor of metalloproteinases (TIMPs) system also contribute to the ECM deposition, not only in the granulation tissue formation stage, but also in the remodeling period. Dysregulation of MMP synthesis and/or disorder of MMP/TIMP balance have been shown in chronic wounds, such as diabetic foot ulcers [29]. MMP-1, a major collagenase, can degrade collagen, while TIMP-1 inhibits the activity of MMP-1 [30]. Although madecassoside could significantly enhance TIMP-1 mRNA expression at the concentration of 3 *μ*M, a more accurate predictor for wound healing, the ratio of MMP-1 to TIMP-1, showed no significant alteration in all four compounds-treated groups. 

Taken together, we draw a conclusion that the two glycosides could facilitate wound healing probably through activating fibroblast, not simply through enhancing cell proliferation. The activation of TGF-*β*/Smad pathway, as evidenced by elevated p-Smad 3 level, raised TGF-*β*_1_ and T*β*RII mRNA levels and downregulated Smad 7 expression, might contribute to the facilitation effect of *C. asiatica* in burn wound healing. Both *in vitro* and* in vivo* findings confirmed that asiaticoside and madecassoside themselves are the active entities. However, the metabolites of the two glycosides, at least the corresponding aglycones, are not, which is contrast to the former presumption proposed by our group and other researchers [18, 20, 25]. Taken together with our unpublished data on pharmacokinetic study of asiaticoside and madecassocide after oral administration, herein we postulate that asiaticoside and madecassoside may have a special absorption and distribution pattern under burn injury condition. More detailed studies are warranted to clarify the action pattern and mechanisms of asiaticoside and madecassoside for wound healing effects.

In conclusion, the present study has identified the active constituents of *C. asiatica* herbs on burn wound healing via both *in vitro* and *in vivo* assays. It is noteworthy that madecassoside possess a more potent therapeutic effect than asiaticoside (*P* = 0.0446 for procollagen type III synthesis *in vitro*, *P* = 0.0057 for wound healing speed and *P* = 0.0491 for wound healing pattern *in vivo*, correspondingly), and the corresponding aglycones of the two glycosides are not the entities with wound healing activities of *C. asiatica*.

## Figures and Tables

**Table 1:** Sequences of primers for RT-PCR.

Target gene	Forward primer	Reverse primer	Size (bp)
Collagen I	CCCACCAATCACCTGCGTACAGA	TTCTTGGTCGGTGGGTGACTCTGA	214
Collagen III	GAGATGTCTGGAAGCCAGAACCAT	GATCTCCCTTGGGGCCTTGAGGT	207
MMP-1	GAGCAAACACATCTGAGGTACAGGA	TTGTCCCGATGATCTCCCCTGACA	185
TIMP-1	TGGGGACACCAGAAGTCAAC	TTTTCAGAGCCTTGGAGGAG	400
TGF-*β*_1_	ACCAACTATTGCTTCAGCTC	TTATGCTGGTTGTACAGGG	197
T*β*RI	TCGTCTGCATCTCACTCAT	GATAAATCTCTGCCTCACG	342
T*β*RII	GCACGTTCAGAAGTCGGTTA	GCGGTAGCAGTAGAAGATGA	493
Smad 7	GCCCTCTCTGGATATCTTCT	GCTGCATAAACTCGTGGTCA	320
*β*-actin	ACATCTGCTGGAAGGTGGAC	GGTACCACCATGTACCCAGG	163
